# CD28 Blockade *Ex Vivo* Induces Alloantigen-Specific Immune Tolerance but Preserves T-Cell Pathogen Reactivity

**DOI:** 10.3389/fimmu.2017.01152

**Published:** 2017-09-20

**Authors:** Barbara Dillinger, Sarah Ahmadi-Erber, Klara Soukup, Angela Halfmann, Silke Schrom, Bernard Vanhove, Peter Steinberger, Rene Geyeregger, Stephan Ladisch, Alexander Michael Dohnal

**Affiliations:** ^1^Tumor Immunology, Children’s Cancer Research Institute (CCRI), St. Anna Kinderkrebsforschung e.V., Vienna, Austria; ^2^Centre de Recherche en Transplantation et Immunologie UMR 1064, INSERM, Université de Nantes, Nantes, France; ^3^Institut de Transplantation Urologie Néphrologie (ITUN), CHU Nantes, Nantes, France; ^4^OSE Immunotherapeutics, Nantes, France; ^5^Institute of Immunology, Center for Pathophysiology, Infectiology and Immunology, Medical University of Vienna, Vienna, Austria; ^6^Clinical Cell Biology, Children’s Cancer Research Institute (CCRI), St. Anna Kinderkrebsforschung e.V., Vienna, Austria; ^7^Department of Pediatrics, Medical University of Vienna, Vienna, Austria; ^8^Center for Cancer and Immunology Research, Children’s Research Institute, Children’s National Medical Center, Washington, DC, United States

**Keywords:** CD28 blockade, tolerance, alloantigen specific, *ex vivo*, graft-versus-host disease, preserved pathogen reactivity

## Abstract

Donor T-cells contribute to reconstitution of protective immunity after allogeneic hematopoietic stem cell transplantation (HSCT) but must acquire specific tolerance against recipient alloantigens to avoid life-threatening graft-versus-host disease (GvHD). Systemic immunosuppressive drugs may abrogate severe GvHD, but this also impedes memory responses to invading pathogens. Here, we tested whether *ex vivo* blockade of CD28 co-stimulation can enable selective T-cell tolerization to alloantigens by facilitating CD80/86-cytotoxic T-lymphocyte-associated protein 4 (CTLA-4) signaling. Treatment of human allogeneic dendritic cell/T-cell co-cultures with a human CD28 blocking antibody fragment (α-huCD28) significantly abrogated subsequent allospecific immune responses, seen by decreased T-cell proliferation and of type 1 cytokine (IFN-γ and IL-2) expression. Allo-tolerization persisted after discontinuation of CD28 blockade and secondary alloantigen stimulation, as confirmed by enhanced CTLA-4 and PD-1 immune checkpoint signaling. However, T-cells retained reactivity to pathogens, supported by clonotyping of neo-primed and cross-reactive T-cells specific for *Candida albicans* or third-party antigens using deep sequencing analysis. In an MHC-mismatched murine model, we tolerized C57BL/6 T-cells by *ex vivo* exposure to a murine single chain Fv specific for CD28 (α-muCD28). Infusion of these cells, after α-muCD28 washout, into bone marrow-transplanted BALB/c mice caused allo-tolerance and did not induce GvHD-associated hepatic pathology. We conclude that selective CD28 blockade *ex vivo* can allow the generation of stably allo-tolerized T-cells that in turn do not induce graft-versus-host reactions while maintaining pathogen reactivity. Hence, CD28 co-stimulation blockade of donor T-cells may be a useful therapeutic approach to support the immune system after HSCT.

## Introduction

Very frequently, complications arise in hematopoietic stem cell transplantation (HSCT). Allogeneic T-cells, recognizing recipient cells as foreign, initiate cytotoxic immune responses known as graft-versus-host disease (GvHD) in up to 80% of patients (1, 2). GvHD can be explained by the prevalence and indefinite persistence of alloantigens in HSCT recipients, resulting in an overwhelming immune reaction unparalleled by any naturally occurring response (3). Also, due to post-HSCT immunodeficiencies caused by delayed cell recovery, patients frequently suffer from infections, even of commensal origin (4, 5). Non-specific immunosuppressive drugs may suppress the onset or severity of GvHD but concurrently facilitate life-threatening infections and disease relapse (6).

Examples of current therapies to reduce the risk for GvHD include pre-transplant serotherapy with thymoglobulin or anti-CD52 antibody and post-transplant immunosuppressive drugs (7). However, these may also prevent graft-versus-leukemia (GvL) effects and increase the incidence of viral or invasive fungal infections up to 70% (8, 9). An alternative therapeutic approach, post-transplant cyclophosphamide treatment, leads to lysis of highly cytotoxic alloreactive haploidentical T-cells (10), but the incidence of acute GvHD still remains high, reaching up to 46% (11), and pathogenic infections still account for 10% mortality after haploidentical HSCT, underscoring the need for improved approaches for GvHD prophylaxis (12).

One innovative approach to abrogate the onset or severity of GvHD is to interfere with T-cell priming mechanisms to skew alloreactive effector T-cell functions toward tolerization and immune regulation *in vivo*. However, interference with CD80/86 co-stimulatory signals through specific blockers, such as cytotoxic T-lymphocyte-associated protein 4 (CTLA-4) fusion proteins, simultaneously inhibits the suppressive activity of regulatory T-cells that crucially depend on CTLA-4 signaling (13). Systemic application of co-stimulation blockers also dampen pathogen-specific reactivity and increase transplant-related mortality (14), diminishing the value of this approach as a viable solution.

To overcome the immunological shut down and side effects associated with co-stimulation blockade administered systemically, adoptive transfer of *ex vivo* allo-tolerized T-cells may be an effective alternative. Allo-tolerized T-cells then potentially confer pathogen-specific immunity to the patients in the immunocompromised post-HSCT period, while not eliciting GvHD against recipient alloantigen. To test this hypothesis, we used a humanized monovalent PEGylated F_ab_ antibody fragment (α-huCD28) blocking human CD28. This molecule acts as a non-crosslinking CD28 antagonist (15, 16) and was chosen because its administration *in vivo* was not associated with severe immunotoxicity, neither in baboons or non-human primates nor in a *trans vivo* NOD/SCID mouse model (15, 17). Moreover, it prevented organ rejection in a preclinical renal transplantation model and downmodulated autoimmunity in collagen-induced arthritis, experimental autoimmune encephalomyelitis, and uveitis models (18–22). Finally, it had shown safety and tolerability in a recently completed phase I clinical trial (23).

**Figure 1:**
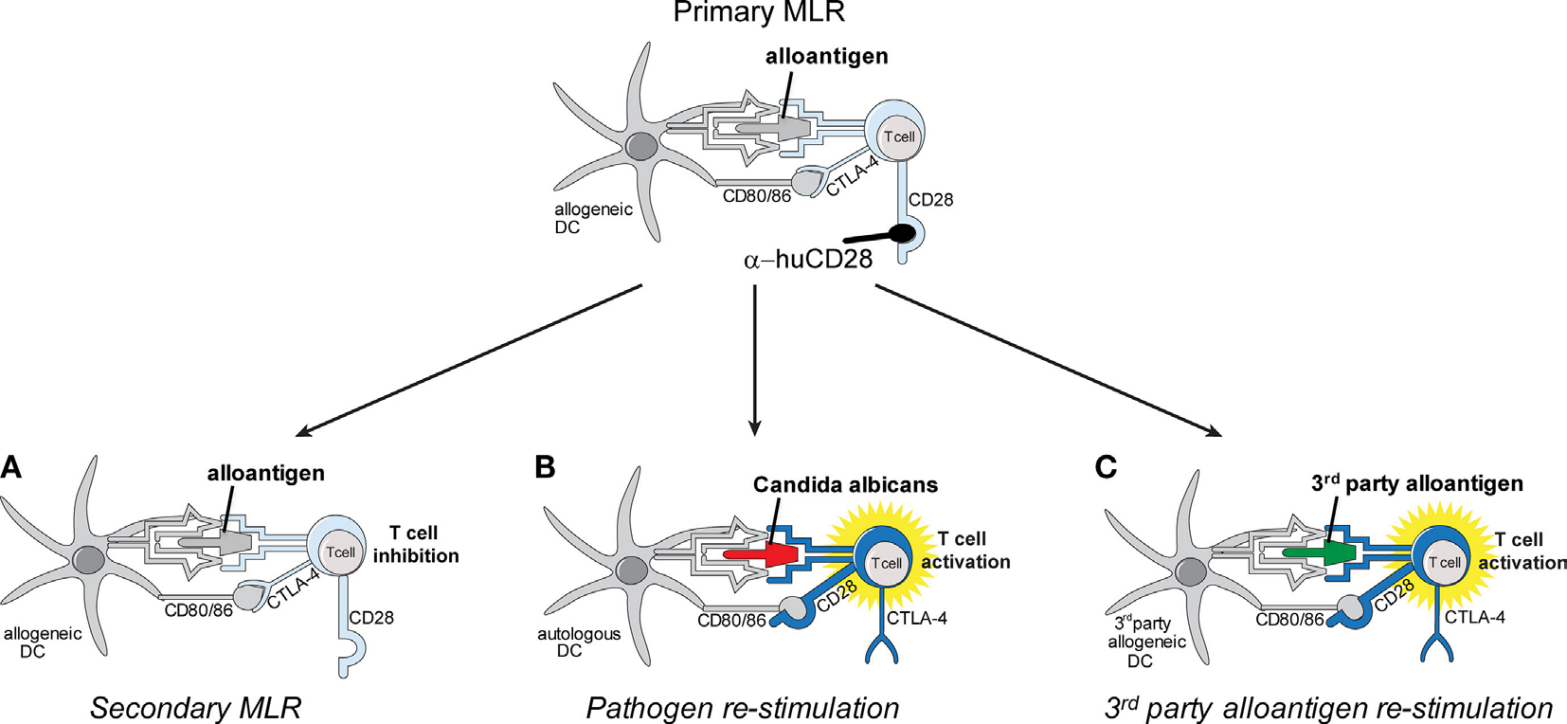
Schema of *in vitro* allo-tolerization and retained pathogen reactivity by α-huCD28-mediated blockade of human T-cells. Alloantigen binding to the respective T-cell receptor (TCR) concurrently with CD28 blockade by α-huCD28 potentially tolerizes human T-cells, while CD80/86 co-stimulatory molecules remain accessible to negative regulators such as cytotoxic T-lymphocyte-associated protein 4 (CTLA-4) (top). Human T-cells are co-cultured with MHC-mismatched human dendritic cells (DCs) presenting alloantigen (primary mixed leukocyte reaction), in the presence of the CD28 blocker α-huCD28. After 7 days of culture, T-cells are washed, rested for 2 days in the absence of α-huCD28, and re-stimulated with **(A)** the same alloantigen (fresh allogeneic DCs), **(B)** *Candida albicans* (autologous DCs), or **(C)** third-party alloantigen (third-party DCs).

We postulated (Figure 1) that *ex vivo* co-culture of T-cells with α-huCD28 could, by blockade of CD28 co-stimulation, induce stable tolerance in T-cells, while permitting these cells to retain pathogen reactivity. Our findings support this possibility.

## Materials and Methods

### Isolation and Differentiation of Human Monocytes

Monocytes were isolated and differentiated into dendritic cells (DCs) as previously described (24) (ethical approval EK 1880/2012 in accordance with the Declaration of Helsinki). On day 6, DCs were stimulated with 50 ng/mL lipopolysaccharide (LPS, *Escherichia coli* O111:B4 LPS, Merck, Darmstadt, Germany) and 10^3^ U/mL human recombinant IFN-γ (Peprotech, Rocky Hill, NJ, USA) for 24 h.

### Isolation of Human T-Cells

Peripheral blood mononuclear cells (PBMCs) were isolated from buffy coats (Rotes Kreuz, Vienna, Austria) and CD3^+^ T-cells were negatively selected by MACS sorting (Miltenyi, Bergisch Gladbach, Germany). For proliferation studies, T-cells were stained with carboxyfluorescein succinimidyl ester (CFSE; Sigma-Aldrich, St. Louis, MO, USA).

### FACS-Based Cell Sorting

CD3^+^ T-cells were sorted (BD FACSAria™ Fusion; BD Biosciences, San Jose, CA, USA) for naive (CD45RA^+^CD45RO^−^) and memory (CD45RA^−^CD45RO^+^) T-cells, excluding dead cells and duplets (Figure S1A in Supplementary Material). The antibodies CD45RA-PE (clone Hl100), CD45RO-BV605 (clone UCHL1; BD Biosciences) were used.

### Tolerance Induction and Re-Stimulation Cultures

As depicted in Figure 1, activated allogeneic DCs and CFSE-stained T-cells were co-cultured for 7 days at a ratio of 1:5 (2 × 10^4^ DCs:1 × 10^5^ Tc) with or without 10 µg/mL α-huCD28 (Figure S1B in Supplementary Material) (15–17, 21, 22, 25) (FR104; OSE Immunotherapeutics, Nantes, France) in RPMI 1640 GlutaMAX™ (Thermo Fisher Scientific) supplemented with 2% Octaplas^®^ (OP, Octapharma, Zurich, Switzerland). T-cells were recovered, rested for 2 days, re-stained with cell proliferation dye 670 (CPD; eBiosciences, San Diego, CA, USA), and counted and re-stimulated again at a ratio of 1:5 (2 × 10^4^ DCs:1 × 10^5^ Tc) with fresh allogeneic DCs (Figure 1A), autologous DCs loaded with UV-inactivated *Candida albicans* (kindly provided by K. Kuchler, MFPL, Vienna, Austria) (Figure 1B), or third-party allogeneic DCs (Figure 1C). A total of 10 or 100 U/mL human recombinant IL-2 (Peprotech) was added to secondary mixed leukocyte reactions (MLRs) to test for the reversibility of tolerance. Different recipient–donor pairs were used as biological replicates for all experiments performed.

### T-Cell Phenotyping and Clonotyping

T-cells were harvested on day 7 (primary MLR) or on days 1, 3, and 7 (secondary MLR). They were stained for cell surface or intracellular markers with the Foxp3/Transcription Factor Staining Buffer Set (eBiosciences) according to the manufacturer’s instructions with the following monoclonal antibodies: CD3-PerCPefl710 (clone SK7), CD25-PeCy7 (clone M-A251), CD28-PeCy7 (clone CD28.2), IFNγ-PE (clone 45.B3), and CTLA-4-PerCPefl710 (clone 14D3) (eBiosciences); CD4-PerCP (clone SK3), CD8 APC-Cy7 (clone SK1) PD-1-BV650 (clone EH12), and CD3-PerCP (clone SK7) (BD Biosciences); IL-2-BV421 (clone MQ1-17H12) (Biolegend, San Diego, CA, USA); and anti-human-F(ab′)_2_-Alexa Fluor 488 (Jackson Immuno Research, West Grove, PA, USA). Cellular proliferation was determined by CFSE/CPD dilution. The percent of proliferated cells was determined by gating on the CFSE^neg^/CPD^neg^ population. The number of proliferating cells was determined as follows: cells were harvested and 10 µL AccuCheck counting beads (Thermo Fisher Scientific) were added shortly before flow cytometric measurement. After determining the total count of CFSE^neg^/CPD^neg^ T-cells and the total bead count, the number of proliferating T-cells was calculated as follows: (total count CFSE^neg^ CPD^neg^/total bead count) × (beads per μL/analyzed volume) = number of proliferated cells/μL. The same calculation was used throughout the manuscript. For calculating the cell number of CTLA-4^++^ or PD-1^+^ CTLA-4^++^ the same calculation approach was used: (total count CTLA-4^++^ or PD-1^+^ CTLA-4^++^/total bead count) × (beads per μL/analyzed volume) = number of CTLA-4^++^ or PD-1^+^ CTLA-4^++^ cells/μL. T-cell phenotyping was performed on a BD LSRFortessa™ (BD Biosciences) flow cytometer and was analyzed with the FlowJo Software (FlowJo LCC).

For clonotyping, DNA was isolated from T-cells harvested on day 7 (primary and secondary MLR from a donor–recipient pair) with the AllPrep DNA/RNA Mini Kit (Qiagen, Hilden, Germany) and analyzed by Adaptive Biotechnologies by T-cell receptor (TCR) β deep sequencing (Illumina HiSeq, Adaptive Biotechnologies, Seattle, WA, USA). Data were normalized by Adaptive Biotechnologies (26) and analyzed using the immunoSEQ analyzer software.

### Immunoblotting

T-cells from co-culture experiments (1 × 10^5^ T-cells seeded) were harvested with one part of 1× PBS and one part of Novex^®^ buffer (Thermo Fisher Scientific) containing 3% dl-dithiothreitol (Sigma-Aldrich). Samples were run on 10% SDS PAGE gels and then blotted on an Amersham protan nitrocellulose transfer membrane (Sigma-Aldrich). Phospho-protein phosphatase 2A (pPP2A) (1:7,000, clone E155; Abcam, Cambridge, UK), protein phosphatase 2A (PP2A) (1:1,000), pAkt (Ser 473, 1:2,000, clone D9E), pAkt (Thr 308, 1:1,000, clone C31E5E), Akt (1:1,000, clone C67E7, all Cell signaling technology, Danvers, MA, USA), and vinculin (clone hVIN-1, 1:40,000 Sigma-Aldrich) protein expressions were determined by immunoblotting of CD3^+^ T-cells at intervals up to 96 h after allogeneic stimulation with DCs. Western blots for pPP2A and pAKt (Ser473 and Thr308) were developed by chemiluminescence imaging using the Super signal west femto maximum sensitivity substrate (Thermo Fisher Scientific) and chemiluminescent detection films (Sigma-Aldrich). Western blots for PP2A, Akt, and vinculin were developed by fluorescence imaging using Goat-anti-mouse IgG Dylight 800 and Goat-anti-rabbit IgG Dylight 800 (both Thermo Scientific) on an Odyssey Fluorescence imager (Licor, Licoln, NE, USA). Western blots were analyzed by densitometry using the ImageJ software.

### Murine Mismatched Transplantation Model

The model consists of bone marrow-derived DC (BMDC) from female BALB/c (H2^d^) mice and CD3^+^ T-cells from female C57BL/6 (H2^b^) mice as described (27). The mice (Charles River Laboratories, Sulzfeld, Germany), aged 6–10 weeks, were maintained under specific pathogen-free conditions at the Biomedical Research Institute, Medical University of Vienna, Austria. All animal experiments were approved by the Austrian Ministry of Science (BMWFW-66.009/0174-WF/V/3b/2015).

Allogeneic BALB/c (H2^d^) bone marrow (BM) was harvested and differentiated to BMDCs as previously described (28) and co-cultured with C57BL/6 (H2^b^) CD3^+^ T-cells for 5 days in the presence or absence of 20 µg/mL α-muCD28 (29) (α28scFv, kindly provided by Pfizer, New York, NY, USA). T-cell proliferation was assessed by CPD dilution and analyzed by flow cytometry (BD LSR Fortessa; BD Biosciences). For immune phenotyping of murine T-cells, the following antibodies were used: CD4-PerCP (clone RM4-5) and CD8-APC-Cy7 (clone 53-6.7, both BD Biosciences). Cell number of proliferated cells was determined as indicated above. Cytotoxicity assays, using murine MC38 tumor cells as targets, were performed as previously described (30). Analysis was done on a BD LSRFortessa™ (BD Biosciences) flow cytometer and was analyzed with the FlowJo Software (FlowJo LCC).

On day −1, BALB/c recipient mice were lethally irradiated by two 4 gray fractions (200 kV X-ray radiation) spaced 4 h apart. On day 0, *ex vivo* treated T-cells were harvested from MLR cultures, washed and sorted for viable cells by FACS. BM from C57BL/6 mice was isolated. A total of 10 × 10^6^ BM cells (i) alone, (ii) together with 0.5 × 10^6^ control (without α-muCD28) T-cells, (iii) T-cells allo-tolerized with α-muCD28, or (iv) non-tolerized (freshly isolated) T-cells were injected intravenously into irradiated BALB/c recipient mice which were monitored daily. A GvHD score was calculated based on four criteria—posture, activity, fur texture, and skin integrity—each scored 0, 1, or 2, as described (31). Upon sacrifice, livers were fixed in 4% paraformaldehyde (Sigma-Aldrich), sectioned, stained with hematoxylin (Dako Agilent Technologies, Glostrup, Denmark) and eosin (Sigma-Aldrich) and histologically analyzed for tissue necrosis, vasodilation, and immune cell infiltration (32). Tissue necrosis was quantified by measuring necrotic areas using Image J software and vasodilation by calculating the mean of the maximum diameters of all vessels in a high power field. Outliers were removed by applying Grubbs’ test (alpha = 0.1). For image acquisition, a Nikon Eclipse 80i microscope with a Nikon DS-Fi1 camera (object lenses 500 μm = 4 × 100 μm = 10×) and NIS-Elements F4.30.00 software were used.

### Statistical Analysis

Data were analyzed by paired, one tailed Student’s *t*-test. *p* ≤ 0.05 was considered statistically significant: ***p < 0.001, **p < 0.005, and *p < 0.05.

## Results

### *Ex Vivo* Blockade of CD28 Persistently Reduces DC-Mediated Alloresponses

**Figure 2:**
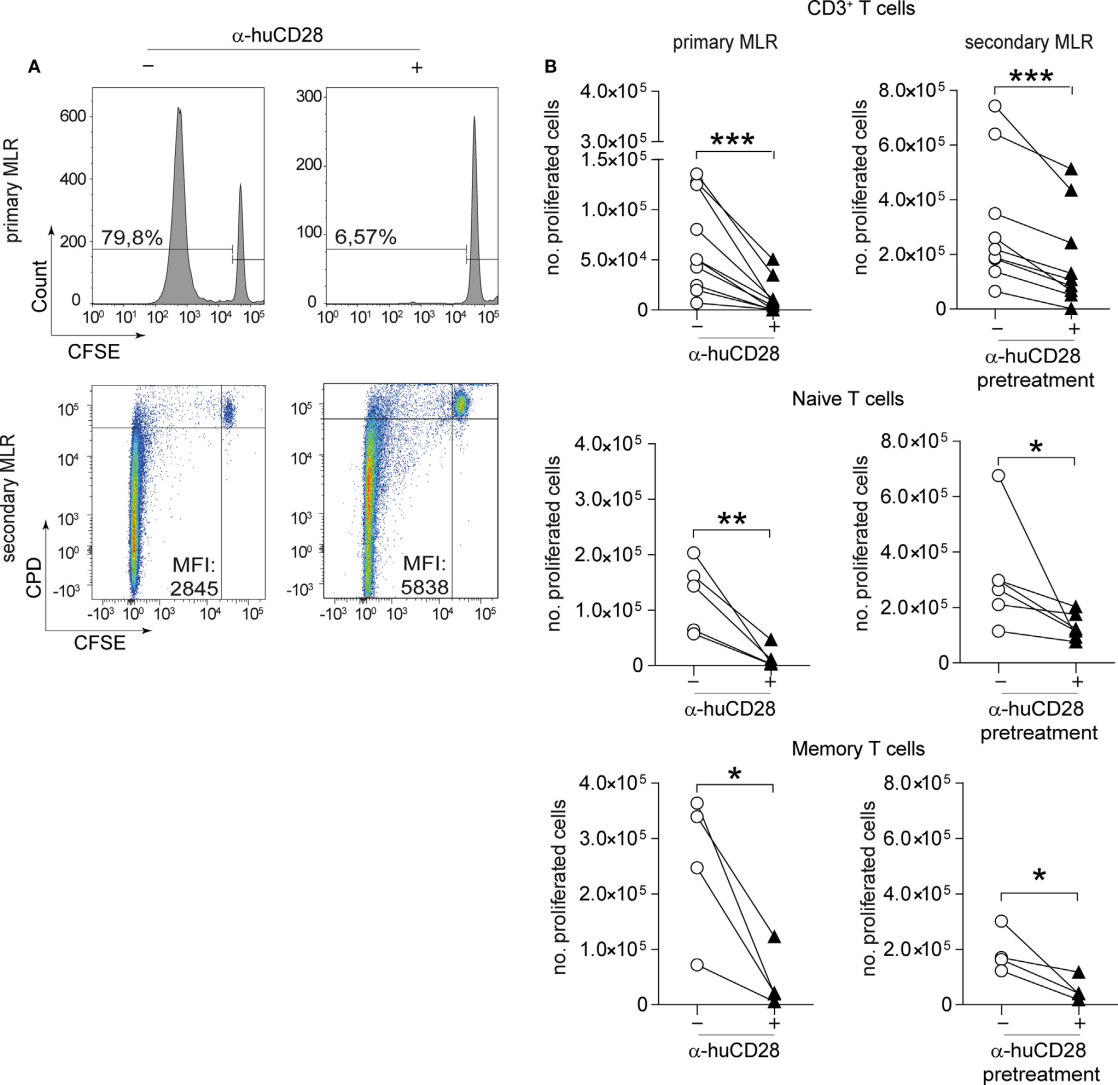
CD28 blockade mediates persistent reduction of alloresponses. Proliferation of human T-cells co-cultured with allogeneic dendritic cells (DCs) *in vitro* in a primary or secondary allogeneic mixed leukocyte reaction (MLR). **(A)** Representative histogram and dotplot analyses of the percentage of carboxyfluorescein succinimidyl ester (CFSE)/CPD dilution and the mean fluorescence intensity of CD3^+^ T-cells in the primary (upper panel) and secondary MLR (lower panel), with (+) or without (−) α-huCD28. Quadrants were adjusted according to unproliferated (CFSE^pos^/CPD^pos^) cells. **(B)** Proliferation (CFSE^neg^/CPD^neg^) of human CD3^+^ T-cells and FACS-sorted naive (CD45RA^+^/RO^−^) or memory (CD45RA^−^/RO^+^) T-cells analyzed by flow cytometry. Proliferation is depicted as number of proliferated cells without (○) or with (▲) α-huCD28 after a 7-day primary MLR (left panel) and a 3-day secondary MLR (right panel). Lines represent results of different biological replicates; points represent the mean of triplicate determinations. Different recipient–donor pairs were used as biological replicates. ***p < 0.001, **p < 0.005, *p < 0.05.

To test the effect of CD28 co-stimulatory blockade on human T-cell responses, we cultured CD3^+^, naive or memory T-cells with or without α-huCD28 (FR104) in a two-stage culture using allogeneic, LPS/IFN-γ-activated DCs (Figure 1, top). This provides a strong CD80/86 co-stimulatory signal, upon binding to CD28 to T-cells (33). In this primary MLR of allogeneic DCs and bulk CD3^+^ T-cells, down modulation of allogeneic T-cell responses was evidenced by greatly reduced proliferation (Figure 2A, upper panel; Figure 2B, left panel), confirming a similar effect previously observed in MLRs using unseparated PBMCs (25).

Upon stimulation with fresh allogeneic DCs from the same donor (secondary MLR, Figure 1A), T-cell proliferation remained downregulated even though cultures were no longer supplemented with α-huCD28. Allo-inhibition was observed three days after secondary stimulation, the mean fluorescence intensity was higher (Figure 2A, lower panel), and the number of proliferated T-cells was still significantly lower (Figure 2B, right panel) in α-huCD28 allo-tolerized, compared to untreated control cultures. Upon incubation with α-huCD28 in primary MLRs, we found that CD28 receptors were occupied on essentially all T-cells (Figure S2A in Supplementary Material). However, staining for the α-huCD28 fragment with a secondary antibody could not detect its presence on cells before secondary MLRs, indicating that CD28 was unoccupied (Figure S2A in Supplementary Material).

Because memory T-cells have been described to be less susceptible to co-stimulation blockade than naive T-cells (34–37), we investigated the effect of CD28 blockade on previously sorted naive (CD45RA^+^, CD45RO^−^) and memory T-cells (CD45RA^−^, CD45RO^+^). Unexpectedly, CD28 blockade reduced alloresponses of both naive and memory T-cells, shown by a significant and sustained decrease in proliferation in primary (Figure 2B, left panel) and secondary (Figure 2B, right panel) MLRs with fresh allogeneic DCs, using multiple different MHC-mismatched donor–recipient pairs. Furthermore, this alloantigen-tolerizing effect in CD3^+^, naive and memory T-cells persisted, up to 7 days (Figure S2B in Supplementary Material). In detail, both CD4^+^ and CD8^+^ T-cell populations showed significantly decreased proliferation upon α-huCD28 treatment in primary as well as in 3- and 7-day secondary MLRs (Figure S3A in Supplementary Material).

IL-2 has been shown to profoundly influence memory responses and to reverse anergy (38, 39). We therefore asked whether IL-2 might also affect α-huCD28 pre-treated T-cells. Although high IL-2 doses overall elevated alloreactivity of naive and memory T-cells, α-huCD28 allo-tolerized T-cells showed up to a fourfold decrease in proliferation after 3 days (Figure S2C in Supplementary Material) and even after extended exposure to IL-2 for up to 7 days (Figure S2C in Supplementary Material). These data suggest that alloreactivity can be persistently reduced by CD28 blockade, even resisting long-term high-dose IL-2 exposure.

### CD28 Blockade Inhibits Th1 Cytokines in Naive and Memory T-Cells in Secondary MLRs

**Figure 3:**
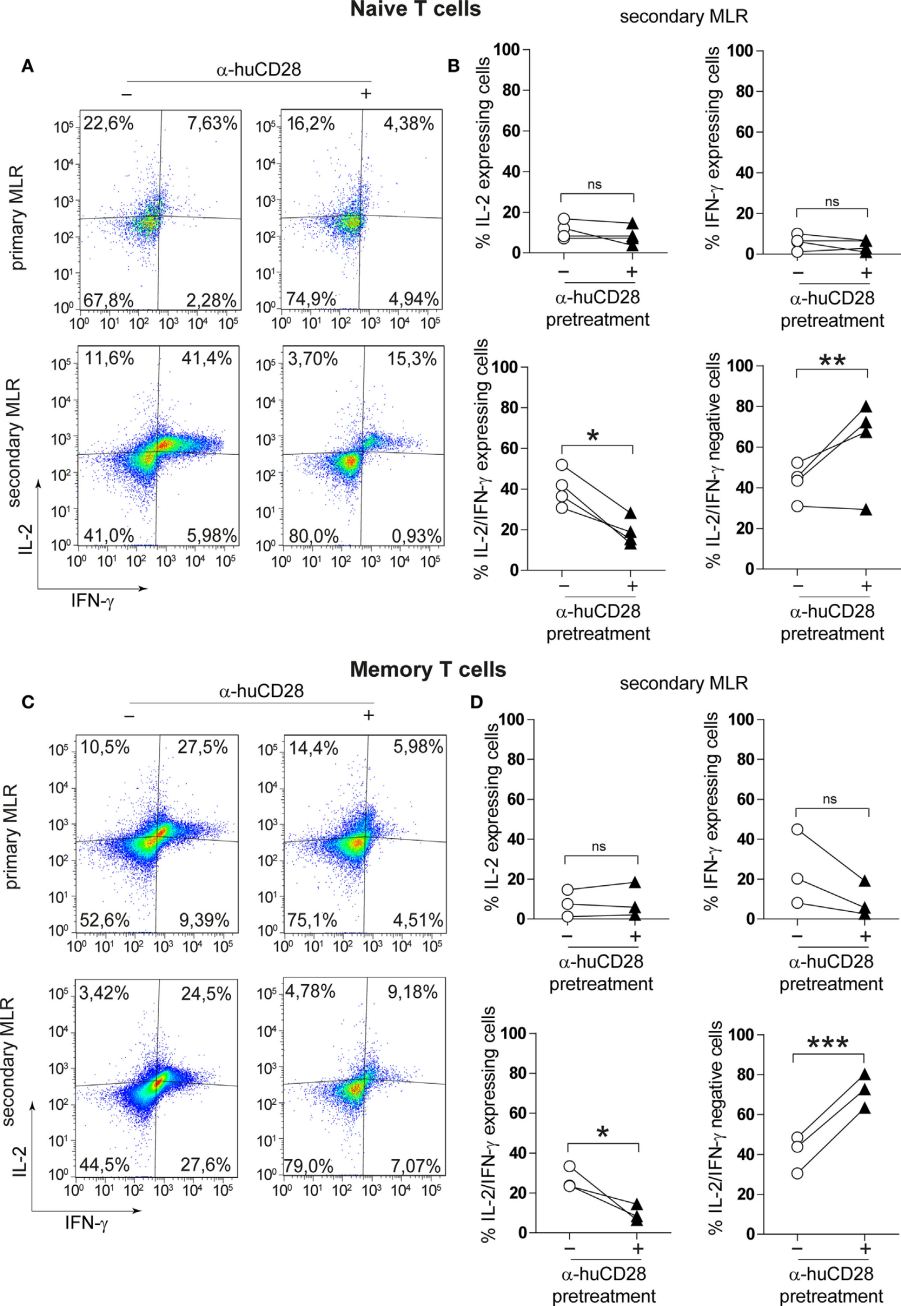
CD28 blockade inhibits Th1 cytokines in secondary allo-mixed leukocyte reactions (MLRs). Th1-specific cytokine expression of FACS-sorted naive and memory T-cells in primary or secondary allo-MLRs. **(A,C)** Representative dotplots show IL-2 and IFN-γ expression of naive **(A)** or memory **(C)** T-cells treated with (+) or without (−) α-huCD28 in a primary and, after discontinuation of CD28 blockade, in a secondary MLR after 24 h. **(B,D)** Percentage of IFN-γ, IL-2, IFN-γ/IL-2 expressing, and IFN-γ/IL-2 negative naive **(B)** or memory **(D)** T-cells, with (▲) or without (○) prior α-huCD28 exposure, in a secondary MLR after 24 h. Pairs of points are the means of triplicate determinations of individual different recipient–donor biological samples. ***p < 0.001, **p < 0.005, *p < 0.05.

Naive as well as memory T-cells can raise strong alloantigen-specific, GvHD-causing immunity associated with Th1 cytokine expression (40, 41). We determined whether CD28 blockade would alter the cytokine expression profile of those T-cell populations. Following 7 days of stimulation of previously unstimulated naive T-cells, we observed only a minor decrease in the number of IL-2 and of IFN-γ/IL-2 expressing T-cells in primary MLRs under CD28 blockade (Figure 3A, upper panel). Although memory T-cells have been described to be less dependent on co-stimulation (34, 37), hence resistant to co-stimulation blockade, the expression of IL-2 and IFN-γ was reduced in primary MLRs in the presence of α-huCD28 (Figure 3C, upper panel). The effect of prior CD28 blockade was highly pronounced upon stimulation with fresh allogeneic DCs in secondary MLRs, seen as a significant decrease in the number of IL-2 and IFN-γ double-positive naive T-cells (Figure 3A, lower panel, Figure 3B) as well as of memory T-cells (Figure 3C, lower panel, Figure 3D). Quadrants were defined according to unstimulated control naive or memory T-cells (Figure S1C in Supplementary Material). These data demonstrate that CD28 blockade leads to stably impaired proliferation and Th1 cytokine production in T-cells.

### CD28 Blockade Increases Checkpoint Signaling in T-Cells

**Figure 4:**
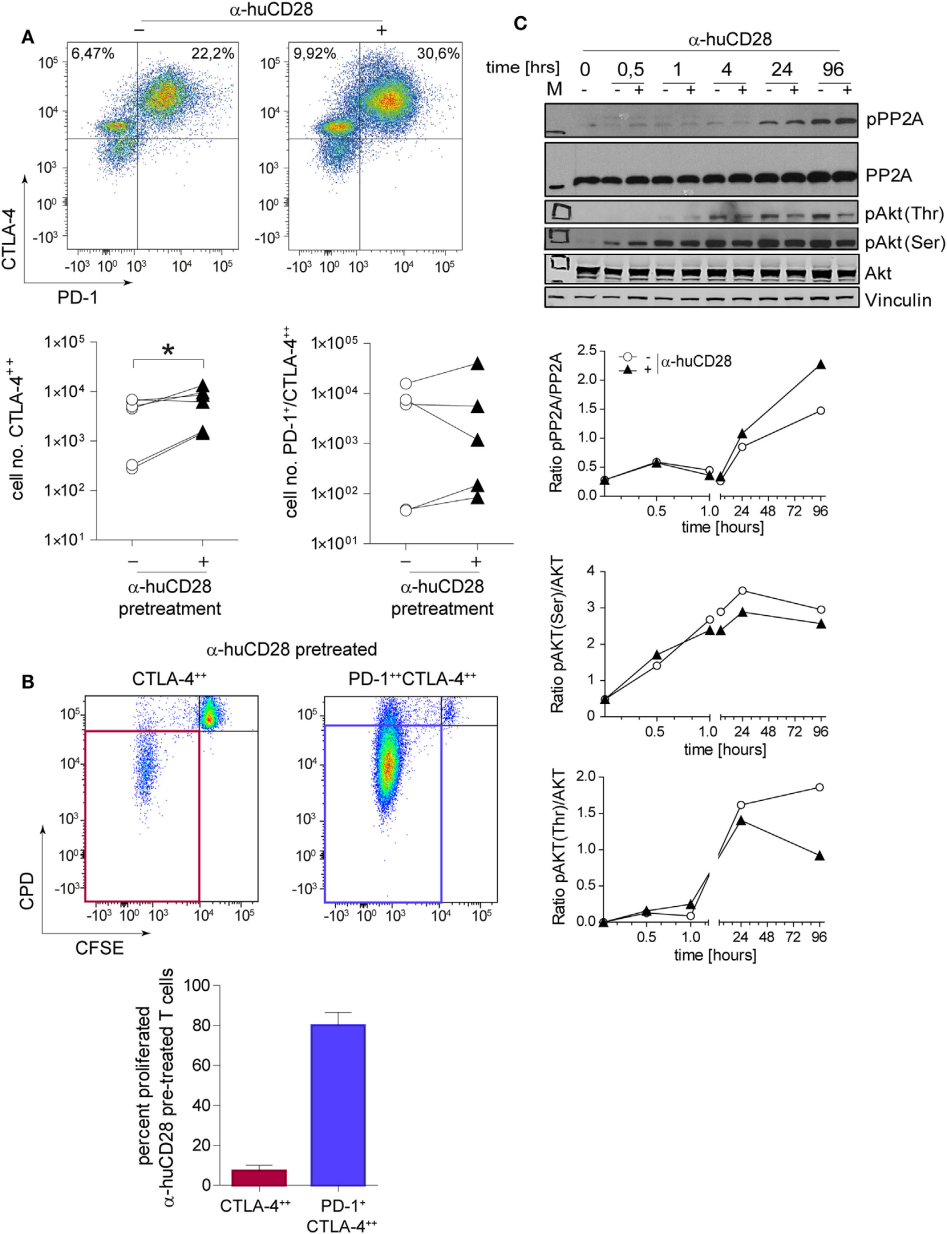
CD28 blockade induces checkpoint signaling. Expression of programmed death 1 (PD-1) and cytotoxic T-lymphocyte-associated protein 4 (CTLA-4) on sorted naive T-cells 24 h after a secondary allogeneic mixed leukocyte reaction (MLR) without (−) or with (+) prior α-huCD28. Cells were gated according to: CD3^+^/live cells/CD4^+^/CTLA-4^++^ or CD3^+^/live cells/CD4^+^/PD-1^+^CTLA-4^++^. CTLA-4^++^ and PD-1^+^CTLA-4^++^ α-huCD28 pre-treated cells were analyzed for cellular proliferation by carboxyfluorescein succinimidyl ester (CFSE)/CPD dilution. **(A)** Representative dotplots of PD-1 and CTLA-4 expression on naive CD4^+^ T-cells in a secondary MLR (day 1). Numbers represent percent within the CD3^+^ cell population. Cumulative results of CTLA-4 and PD-1 expressions of naive T-cells with (▲) or without (○) prior α-huCD28 after a 1 day secondary allo-MLR. Points represent the mean of replicates from different recipient–donor pairs. **(B)** T-cells with prior CD28 blockade, highly expressing CTLA-4 or PD-1/CTLA-4, were analyzed for proliferation (CFSE^neg^/CPD^neg^, proliferated). Representative dotplots of CFSE/CPD staining are shown; bar graphs show the cumulative results of percent proliferated T-cells. **(C)** CD3^+^ T-cell lysates from a primary MLR with (+) or without (−) α-huCD28 for 0–96 h were examined by Western blot to detect phospho-protein phosphatase 2A (pPP2A), total protein phosphatase 2A (PP2A), Akt phosphorylated on threonine 308 (pAkt Thr) or serine 473 residues (pAkt Ser), total Akt, and vinculin as a control. For densitometric analysis (ratio of phosphorylated/total protein) the ImageJ program was used. *p < 0.05.

Immune checkpoint expression on T-cells is associated with a tolerogenic T-cell phenotype (42, 43). Therefore, we assessed CTLA-4 and PD-1 levels of CD28-blocked allogeneic T-cells after secondary alloantigen stimulation. α-huCD28 pre-treatment of MLR cultures resulted in a significant increase in CTLA-4 expression on alloantigen-primed naive T-cells, with a tendency toward increased PD-1/CTLA-4 co-expression (Figure 4A). Furthermore, CTLA-4-expressing cells essentially did not proliferate, whereas PD-1/CTLA-4 co-expressing cells did (Figure 4B). Therefore, we deduced that CD28 blockade caused either cellular arrest or substantial exhaustion of previously proliferating allo-stimulated naive T-cells (44, 45).

Blockade of CD28 by α-huCD28 is proposed to spare the CD80/86-CTLA-4 signaling axis (Figure 1, top) (16). We therefore investigated both the signaling molecule PP2A that releases CTLA-4 upon phosphorylation and the key downstream molecule Akt (protein kinase B) (46), which is then bound by pPP2A, preventing its (Akt) own phosphorylation. In our experiments, PP2A activity, induced by its phosphorylation, was enhanced while phosphorylation of Akt, both on Thr and Ser residues, was decreased upon stimulation with alloantigen under CD28 blockade, compared to untreated cultures (Figure 4C). Decreased phosphorylation on Thr residues was more pronounced than on Ser residues, which is in line with previous observations that PP2A preferentially targets Thr phosphorylation sites (47, 48). These results combined are presumably the cause of inhibited growth and proliferation, as we observed for CTLA-4 positive allo-tolerized T-cells (Figure 4B). In summary, α-huCD28 allo-tolerized T-cells showed increased expression of CTLA-4 and PD-1 consistent with enhanced phosphorylation of the CTLA-4 inhibitor PP2A and its downstream molecule Akt, suggesting development of a tolerogenic T-cell phenotype.

### T-Cells Retain Proliferative Capacity upon Pathogen Exposure Despite Effective Allo-Tolerization

**Figure 5:**
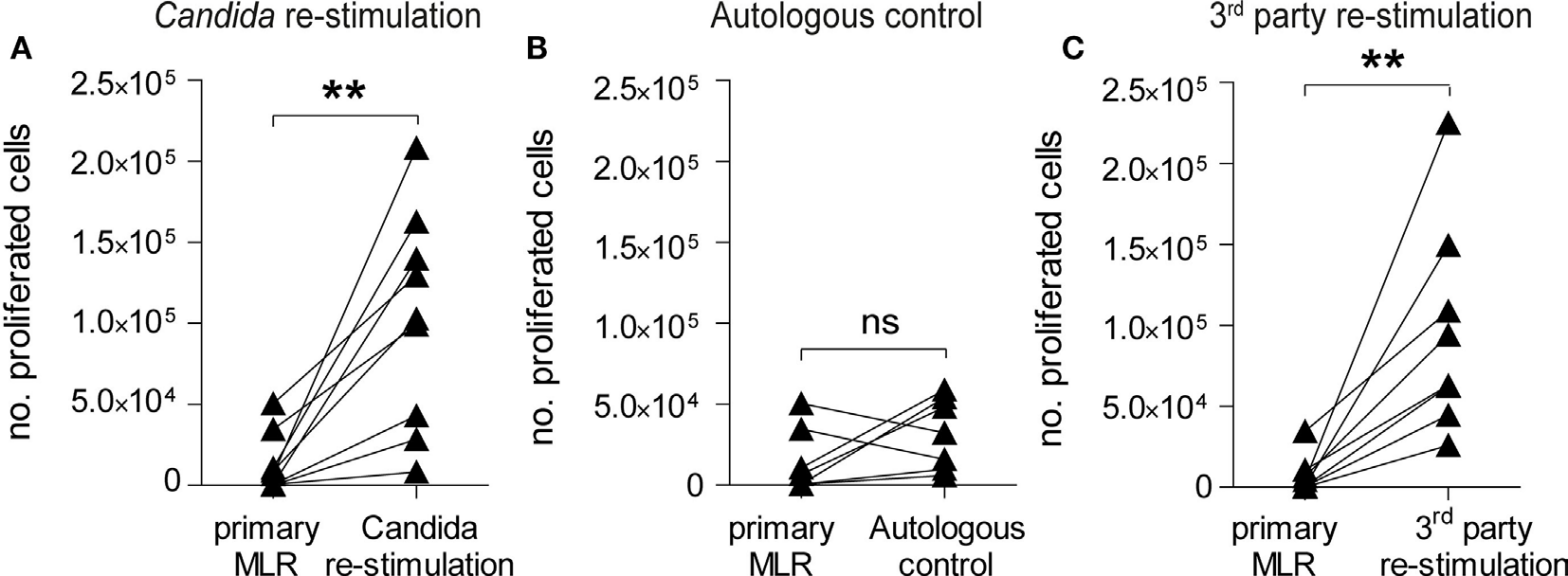
CD28-tolerized T-cells retain pathogen reactivity. Proliferation of human CD3^+^ T-cells after re-stimulation with *Candida albicans* or foreign third-party alloantigen. **(A–C)** Data points for primary mixed leukocyte reactions (MLRs) are derived from Figure 2B (+ α-huCD28) for comparison. The number of proliferated cells in primary MLRs ranged from 1 × 10^2^ to 9 × 10^3^. T-cell proliferation, shown as number of proliferated cells, was analyzed by flow cytometry after a 7-day re-stimulation with **(A)** autologous dendritic cells with *C. albicans*, **(B)** without *C. albicans* as a control for possible antigen-independent proliferation, or **(C)** with foreign third-party alloantigen. Each point is the mean of triplicate determinations of different recipient–donor pairs used as biological replicates. **p < 0.005; ns = not significant, *p* > 0.05.

Infusion of donor lymphocytes with retained pathogen reactivity after alloantigen-tolerization can be pivotal for HSCT patients upon pathogen encounter (49, 50). To test the reactivity of α-huCD28 allo-tolerized T-cells against *C. albicans*, washed CD3^+^ T-cells recovered from primary MLRs with α-huCD28 were re-stimulated with autologous DCs loaded with UV-inactivated *C. albicans* (Figure 1B). This resulted in significant T-cell expansion after allo-tolerization upon re-stimulation with *C. albicans* (Figure 5A). In unstimulated cultures (autologous DCs without *C. albicans*), T-cells did not show significant proliferation (Figure 5B), suggesting that the proliferation was *Candida* antigen-specific and excluding an antigen-independent mechanism for the observed proliferative response to *Candida*. Furthermore, T-cell re-stimulation with foreign third-party allogeneic DCs (Figures 1C and 5C) also resulted in significant expansion after allo-tolerization, confirming specificity of allo-tolerization against primary alloantigen. The same effect was observed when analyzing proliferative responses of CD4^+^ and CD8^+^ T-cells to foreign antigen (Figure S3B in Supplementary Material). Thus, despite effective and specific tolerization to the primary alloantigen, CD28 blockade did not impede *C. albicans* or third-party-specific reactions in re-stimulation cultures.

### Pathogen-Reactive T-Cell Clones Expand Despite Effective CD28-Mediated Allo-Tolerization

To investigate, whether clonal expansion of allo-tolerized T-cells to unrelated antigen derived from unique *C. albicans* and third-party reactive clones, or from cross-reactive clones, we sequenced TCRβ chains of allo- and *Candida*-antigen-reactive T-cells and searched for cross-reactive and unique clones in the T-cell repertoire, the latter representing highly specific foreign antigen reactive clones.

**Figure 6:**
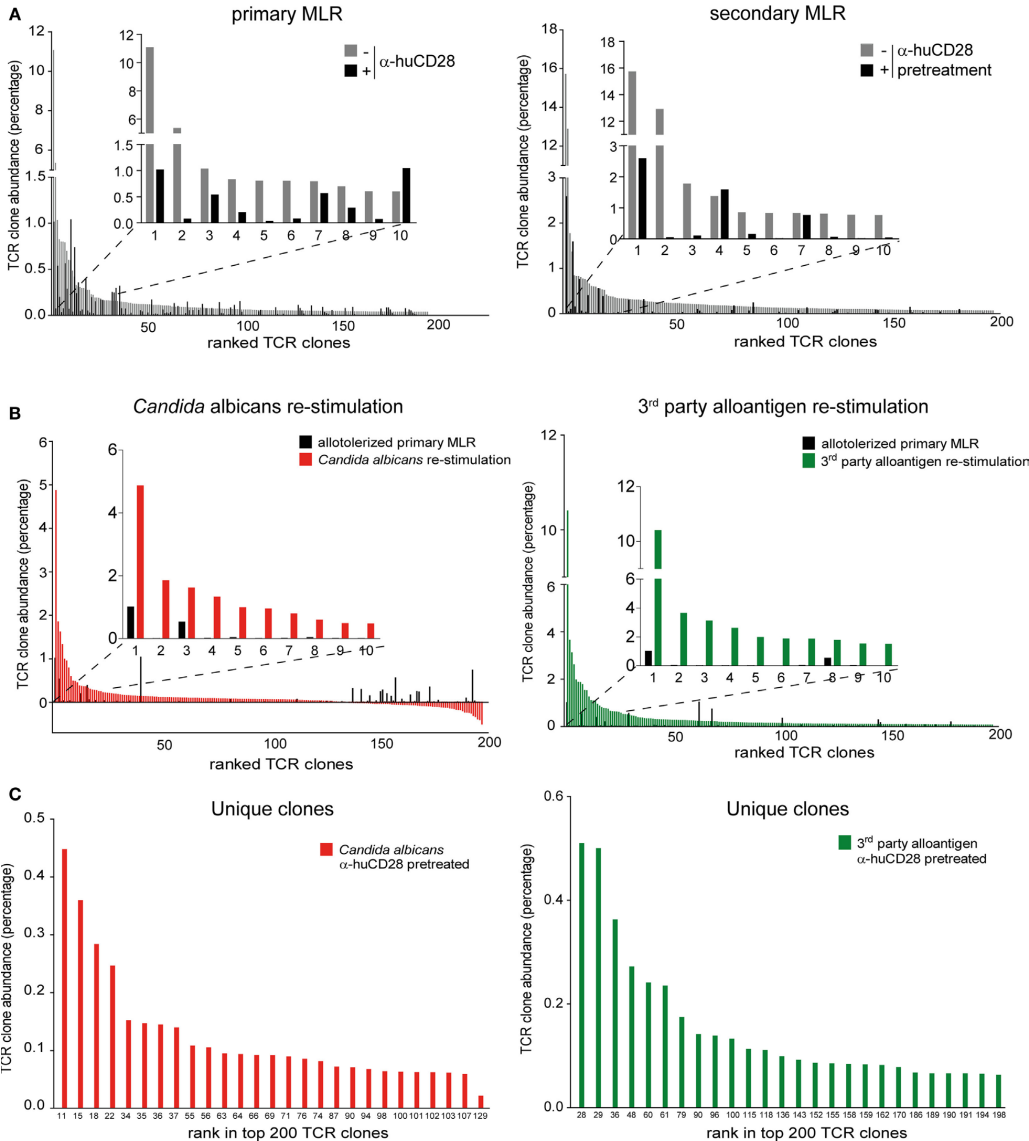
Expansion of allo- versus pathogen-reactive T-cell clones after α-huCD28-mediated blockade. Clonal frequency of CD3^+^ T-cell cultures from a primary recipient–donor pair mixed leukocyte reaction (MLR) and re-stimulation with either 1st party alloantigen, *Candida albicans*, or third-party alloantigen, as analyzed by deep TCRβ sequencing. **(A)** Expansion of T-cell clones in a primary MLR without (gray bars) or with α-huCD28 (black bars) and a secondary MLR in the absence of α-huCD28. Top 200 clones are ranked from their lowest to highest frequency in cultures without α-huCD28. The top 10 clones are represented individually in all graphs. **(B)** Clonal expansion after allo-tolerization and re-stimulation with *C. albicans* or third-party alloantigen. The frequency of antigen-independent expanded T-cell clones (T-cells stimulated with autologous DCs without *C. albicans*) was subtracted from the Candida-specific clonal expansion. The top 200 clones were ranked from high to low abundance in *C. albicans* (red bars) or third-party alloantigen (green bars) re-stimulation cultures and compared to allo-tolerized primary MLRs (black bars). For an in depth investigation, the top 10 most expanded clones are represented individually in all graphs. Clone numbers 2 and 4–10 (left graph) and 2–7 and 9–10 (right graph) are present in primary MLRs, but in low numbers ranging from 0.004 to 0.039%. **(C)** Unique clones expanded upon stimulation with *C. albicans* (red bars) or third-party alloantigen (green bars) but not present in secondary MLRs with 1st party alloantigen. Clones are ranked from high-to-low abundance in the top 200 clonal repertoire (numbers at the bottom of the figure).

First, to confirm inhibition of alloreactive clones upon CD28 blockade, we compared the clonal frequency of expanding T-cell clones in primary and secondary MLRs. Vigorous clonal expansion in the primary MLR without co-stimulation blockade was observed, as expected (51). However, 9 of the top 10 most expanded alloreactive T-cell clones were suppressed by up to 90% upon blockade of CD28 (Figure 6A, left graph; Figure S4A in Supplementary Material left table). Eight of these same clones remained up to 80% inhibited in a secondary MLR in the absence of CD28 blockade (Figure 6A, right graph; Figure S4A in Supplementary Material right table). In total, we observed persistent inhibition of 192 of the 200 top-ranked alloreactive T-cell clones.

After allo-tolerization in primary MLRs in the presence of α-huCD28 and then re-stimulation with *C. albicans*, we detected 146 enriched T-cell clones (Figure 6B, left graph; Figure S4B in Supplementary Material left table). To exclude antigen-independent driven expansion, we subtracted the frequency of T-cell clones that non-specifically expanded in control cultures without *Candida* from the frequency of clones in *Candida* stimulated cultures. A similar pattern was observed upon re-stimulation with third-party alloantigen: 192 of the top 200 third-party alloantigen-enriched T-cell clones expanded up to 10-fold, compared to primary allo-tolerized cultures (Figure 6B, right graph; Figure S4B in Supplementary Material right table). Since the dominant clone (CASSPDLNSPLHF) was also the one most enriched under first party, *Candida*, and third-party alloantigen re-stimulation (Figures S4A,B in Supplementary Material), we selected for unique T-cell clones exclusively enriched after *Candida* or third-party alloantigen and not present upon first-party alloantigen re-stimulations. Indeed, we detected 27 unique and 110 cross-reactive *Candida* and 26 unique and 174 cross-reactive third-party-specific expanded T-cell clones (Figure 6C). Although the abundance of these unique clones was low (up to 0.45% for *Candida* and 0.51% for third-party re-stimulation; Figure S4C in Supplementary Material), they expanded more than 200-fold upon foreign antigen re-stimulation compared to their abundance of <0.002% in the original T-cell pool before allo-tolerization.

In summary, we confirmed *C. albicans* and third-party-specific clonal expansions after CD28 allo-tolerization that arose from neo-primed or cross-reactive T-cells. This suggests that the *ex vivo* treatment we explored does not eliminate responsiveness to new antigen exposure, even though response to primary alloantigen that could cause GvHD is markedly reduced.

### T-Cells Allo-Tolerized by α-muCD28 *Ex Vivo* Do Not Induce GvHD in Mice

**Figure 7:**
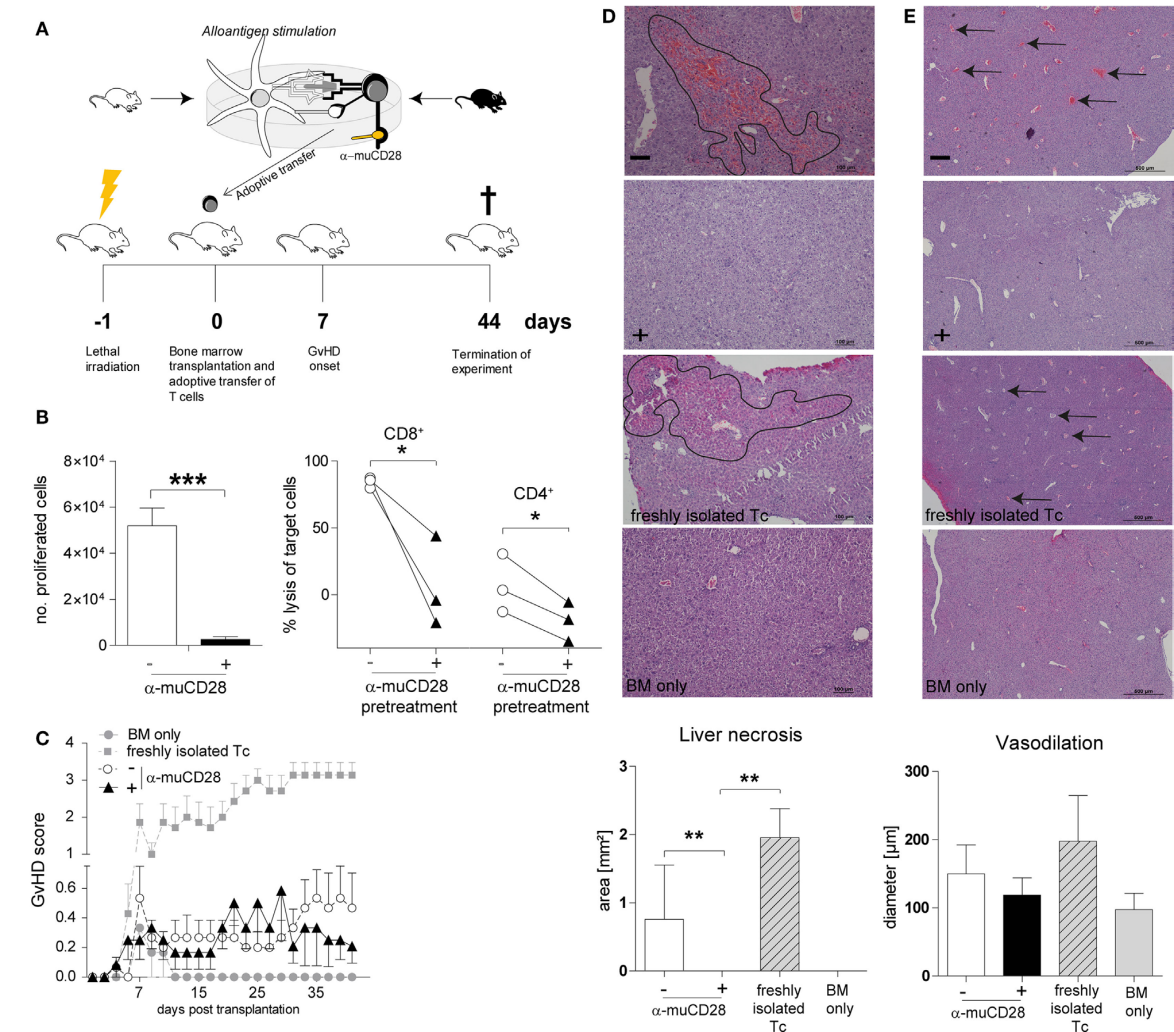
*Ex vivo* co-stimulation blocked T-cells do not induce graft-versus-host disease (GvHD) in an MHC-mismatched murine model. Experimental schema **(A)**: T-cells isolated from C56BL/6 spleens were co-cultured with BALB/c dendritic cells (DCs) for 5 days with or without α-muCD28. Lethally irradiated BALB/c mice were transplanted with C56BL/6 bone marrow (BM) and FACS-sorted viable T-cells from mixed leukocyte reaction (MLR) cultures. Mice were monitored for signs of GvHD for 44 days. **(B)** T-cell proliferation: depicted by the number of proliferated C56BL/6 T-cells upon stimulation with BALB/c DCs together with (+) or without (−) α-muCD28 after a 5-day MLR (left graph), before infusion; cytolytic activity: cytotoxicity of alloreactive CD8^+^ and CD4^+^ T-cells (right graph) against MC38 target cells after 21 h is shown. **(C)** GvHD score of BALB/c mice transplanted with C57BL/6 BM alone (●, *n* = 6) or together with non-tolerant (freshly isolated) T-cells (■, *n* = 7), or T-cells without (○, *n* = 15) or with prior α-muCD28 treatment *ex vivo* (▲, *n* = 12). **(D)** H&E staining of BALB/c livers, necrotic lesions are indicated by black borders, 10× magnification. Necrosis was assessed by quantifying the affected areas using the ImageJ program (− α-muCD28 *n* = 11, + α-muCD28 *n* = 11, freshly isolated Tc *n* = 5, and BM only *n* = 6). Outliers were removed by applying Grubbs’ test (alpha = 0.1). The sum of the areas of all measurable lesions in a high power field is depicted. Bars represent 100 µm. **(E)** Dilated blood vessels are indicated by black arrows, 4× magnification. Vasodilation was quantified as the mean of all vessel diameters in a high power field (− α-muCD28 *n* = 11, + α-muCD28 *n* = 10, freshly isolated Tc *n* = 4, BM only *n* = 4). Outliers were removed by applying Grubbs’ test (alpha = 0.1). Bars represent 500 µm. Image acquisition in **(D,E)** was done with a Nikon Eclipse 80i microscope with a build on camera Nikon DS-Fi1. Magnification of the object lenses was 500 μm = 4×, 100 μm = 10×. NIS-Elements F4.30.00 software was used for image acquisition. Data shown comprise two separate experiments, scored in a blinded fashion independently by two observers. **p < 0.005, *p < 0.05.

Taken together, our findings using human alloreactive T-cells suggest that it is possible to selectively induce tolerance by CD28 blockade *ex vivo* and that this can persist in secondary MLRs *in vitro*. To assess the *in vivo* significance of these findings, we tested the effect of CD28 co-stimulation blockade, once again *ex vivo*, on allogeneic donor T-cells in a murine MHC-mismatched GvHD model (Figure 7A). This model has been previously described to be suitable for *in vivo* evaluation of co-stimulation blockade (15, 52). We co-cultured donor T-cells with allogeneic DCs from recipient mice in the presence of α-muCD28, a single chain fragment blocking murine CD28 (29). As an *in vitro* control for inhibited proliferation by CD28 blockade, we showed that α-muCD28 allo-tolerized T-cells proliferated significantly less than did T-cells not exposed to α-muCD28 before infusion (Figure 7B, left graph). CD8^+^ alloreactive T-cells, known to have high cytolytic capacity in GvHD (53), and also CD4^+^ alloreactive T-cells, showed significantly reduced cytotoxicity when CD28 had been blocked (Figure 7B, right graph). Additionally, we showed that treatment of murine DC/T-cell cultures with α-muCD28 increased the frequency of regulatory T-cells on day 5 in primary MLRs (Figure S5 in Supplementary Material).

We injected equal numbers of FACS-sorted, living, *ex vivo* allo-tolerized, washed T-cells intravenously into lethally irradiated, BM-transplanted recipient mice. GvHD was monitored for 44 days. We observed that infused T-cells that were pre-treated with α-muCD28 did not induce GvHD, as shown by a low GvHD score (Figure 7C). While we observed only a minor difference in the GvHD score in mice receiving T-cells that were pre-treated with α-muCD28 (▲) versus not pre-treated (○) (Figure 7C), the hepatic histological findings were striking. The liver, one of the main target organs in GvHD (54, 55), was not affected by the α-muCD28-modulated T-cells (Figures 7D,E); there were significantly less necrotic lesions (Figure 7D) and a marked decrease of vasodilation (Figure 7E) in livers of mice treated with α-muCD28-modulated T-cells (+) compared to control mice injected with non-tolerized (−) or freshly isolated T-cells. Histologically, livers of mice treated with α-muCD28-modulated T-cells showed no signs of organ damage and resembled the livers of mice that received only BM and no added T-cells (in which no damage was expected) (27). We did not detect differences in immune cell infiltration among the treatment groups (data not shown). These findings show that alloreactive T-cells, blocked for CD28 *ex vivo*, do not induce GvHD in mice.

## Discussion

Induction of immune tolerance in human T-cells is a subject of great experimental and clinical interest (56). Activation of T-cells requires not only antigenic signaling through the TCR but also a co-stimulatory signal delivered *via* the CD28 receptor (57). The central role of delivery of this co-stimulatory signal by CD28 receptor ligation is to launch antigen-specific effector functions in combination with TCR occupancy (25). To deprive T-cells of co-stimulation and prevent GvHD in HSCT patients, attempts to tolerize T-cells against alloantigens have included the development of inhibitors, such as those blocking CD28 signaling. One approach has been to use a CTLA-4 fusion protein [e.g., Abatacept (14)] that competes with CD28 for CD80/86 binding (58) to inhibit CD80/CD86-mediated CD28 ligation. Exposure of donor PBMCs to Abatacept prior to haploidentical HSCT did reduce alloresponses but was apparently not selective only for alloreactive T-cells, since 4 of the 12 patients developed severe bacterial and fungal infections (59) and 5 of 11 patients developed cytomegalovirus reactivations (60) after infusion. Belatacept, a second-generation CTLA-4 fusion protein, was shown to allotolerize human T-cells without losing reactivity to Epstein–Barr virus *in vitro* (61).

An alternative approach is to block CD28. This could leave the inhibitory CD80/CD86-CTLA-4 pathway intact, unlike the effect of CTLA-4 fusion proteins. Here, we studied a novel F_ab_ fragment that blocks CD28, specifically asking whether *ex vivo* binding to donor T-cells, together with stimulation with allogeneic DCs, can induce stable tolerance in T-cells. We found that indeed α-huCD28-mediated CD28 blockade *ex vivo* drives human T-cells toward a stable unresponsive state, selectively against recipient alloantigens. The decreased T-cell proliferation not reversed by IL-2 addition, strongly reduced production of Th1 cytokines even after secondary alloantigen stimulation, and retained pathogen reactivity support this conclusion. Further evidence for successful tolerization was derived from our *in vivo* data, where adoptive transfer of α-muCD28 tolerized murine T-cells into MHC-mismatched mice did not induce GvHD and liver damage. Moreover, blockade of CD28 increased the inhibitory CTLA-4 immune checkpoint signaling, suggesting persistence of the tolerized state of alloantigen-reactive T-cells. As it was shown that CD28 blockade does not interfere with Treg function while blocking alloreactive effector T-cells (13), we as well could show an increase of regulatory T-cells in murine DC/T-cell cultures upon α-muCD28 treatment. The increase in immunosuppressive molecules on human T-cells pre-treated with α-huCD28 could thus hint toward an increased presence of human regulatory T-cells as shown by Poirier et al. (15). Tolerization was also selective in that pathogen-specificity *in vitro* after allo-tolerization was retained. This was confirmed by deep TCR sequencing showing single T-cell clone enrichment upon stimulation with fungal and third-party antigens. Although alloantigen-specific proliferation was not completely abrogated in secondary MLRs, proliferation was still up to 50% lower compared to unblocked cultures. Hence, we conclude that after infusion of CD28-blocked donor T-cells, residual alloreactivity but also T-cells remaining responsive to minor histocompatibility antigens potentially support beneficial pathogen defense and GvL effects (62). Immune reactions driven by minor histocompatibility antigens require an MHC match between recipient and engrafted donor immune system to make tumors or infected cells visible to donor T-cells. This may happen in haploidentical transplant settings, which was not facilitated in our MHC mismatch mouse model. Remaining pathogen and tumor reactivity of CD28-blocked donor T-cells need to be investigated in further studies using haploidentical murine transplantation models.

Down modulation of proliferation and of Th1 cytokines, impaired cytotoxicity, and upregulation of immune inhibitory pathways in T-cells characterize immune tolerant conditions as being an exhausted T-cell phenotype (63, 64). We observed this T-cell phenotype after exposure to α-huCD28. Proliferation and Th1 cytokine production were slightly restored after discontinuation of CD28 blockade and secondary alloantigen stimulation. However, residual proliferating T-cells showed elevated PD-1 levels, an indicator of exhaustion (64). Upregulation of PD-1 and CTLA-4 had also been shown in kidney biopsies of baboons treated with α-huCD28 systemically (19). The suggestion that T-cells are permanently inhibited and do not reactivate upon alloantigen challenge was further supported by our findings in the GvHD *in vivo* model.

Considering signaling pathways involved in CTLA-4 mediated regulatory responses, PP2A is a key molecule initiating such inhibitory cascades. In resting T-cells, PP2A is unphosphorylated and bound to CTLA-4 intracellularly (65, 66), preventing CTLA-4 from migrating to the surface to act as an immunoinhibitory receptor that competes with CD28 for CD80/86 binding. Concurrently, Akt performs its normal regulatory function of initiating cell proliferation and survival (67). Upon T-cell activation, however, PP2A becomes phosphorylated and releases CTLA-4 while binding to Akt, inhibiting its phosphorylation and preventing cell cycle progression. In the case of GvHD, however, CTLA-4-mediated T-cell inhibitory processes are inadequate to suppress the cellular response *in vivo*. We suggest that blockade of CD28 (such as with α-huCD28) could intensify such immune inhibition. This hypothesis is supported by our findings of increased CTLA-4, increased phospho-PP2A, and decreased downstream Akt activity of *ex vivo* tolerized T-cells. Overall, the findings support the concept that T-cell tolerance induced by CD28 blockade, leaving CTLA-4 accessible as a negative regulator (15) (model in Figure 1), leads to persistent alloantigen-specific immunological exhaustion.

Further evidence for specificity is derived from clonal analysis. We documented (a) the reduction of alloreactive clones by α-huCD28 treatment as well as (b) their persistent downmodulation after secondary allo-MLRs and importantly (c) maintenance of pathogen and third-party reactive, unique clones despite prior CD28 blockade. Upon re-stimulation with foreign microbial or third-party derived antigen, the TCR repertoire clearly shifted toward overrepresented *Candida* or third-party-specific clones, of which the frequencies were low or below detection limit in primary MLRs. This suggests a potent induction of antigen-specific clones, while the expansion of alloreactive clones is still retained.

Thus, the *ex vivo* approach to tolerize T-cells through α-huCD28-mediated blockade not only reduces donor-derived alloresponses but also concurrently, in contrast to systemic application of co-stimulation blocking agents, spares pathogen-specific donor lymphocytes.

With respect to a clinical application the question is whether selective tolerance can be accomplished in HSCT patients—patients in whom lack of immunological memory makes them susceptible to high mortality from opportunistic infections (68, 69). Specifically, since adoptive transfer of *ex vivo* α-muCD28-blocked donor T-cells decreased GvHD pathophysiology in mice, can *ex vivo* α-huCD28-modified donor T-cells ameliorate GvHD in humans? Could sufficient numbers of tumor-free recipient DCs be generated prior to chemotherapy? If so, allo-tolerized T-cells could be given as prophylactic therapy concurrently with the graft in HSCT for various malignancies, including leukemias. Standardization of cell preparation techniques may help in yielding clinical grade T-cells that maintain reactivity against pathogens and tumor cells and, therefore, be superior to currently available therapies that induce systemic immune suppression.

## Ethics Statement

This study was carried out in accordance with the recommendations of “Tierversuchsgesetz 2012, BGBl. I Nr. 114/2012, Austrian Ministry of Science.” The protocol was approved by the “Austrian Ministry of Science (BMWFW-66.009/0174-WF/V/3b/2015).”

## Author Contributions

BD designed and performed experiments, analyzed data, and wrote the manuscript. SA-E designed and performed experiments and analyzed data. KS, AH, and SS performed experiments. BV, PS, and RG revised the manuscript. SL wrote the manuscript and AD designed experiments and wrote the manuscript.

## Conflict of Interest Statement

BV is chief operating officer and shareholder of OSE Immunotherapeutics. All other authors declare that the research was conducted in the absence of any commercial or financial relationships that could be construed as a potential conflict of interest. The reviewer PC and handling Editor declared their shared affiliation.
