# Supplementary figures and images for: CD28 Blockade Ex Vivo Induces Alloantigen-Specific Immune Tolerance but Preserves T-Cell Pathogen Reactivity
Source: Front Immunol. 2017 Sep 20;8:1152. doi: 10.3389/fimmu.2017.01152 (PMC5611377; doi:10.3389/fimmu.2017.01152)

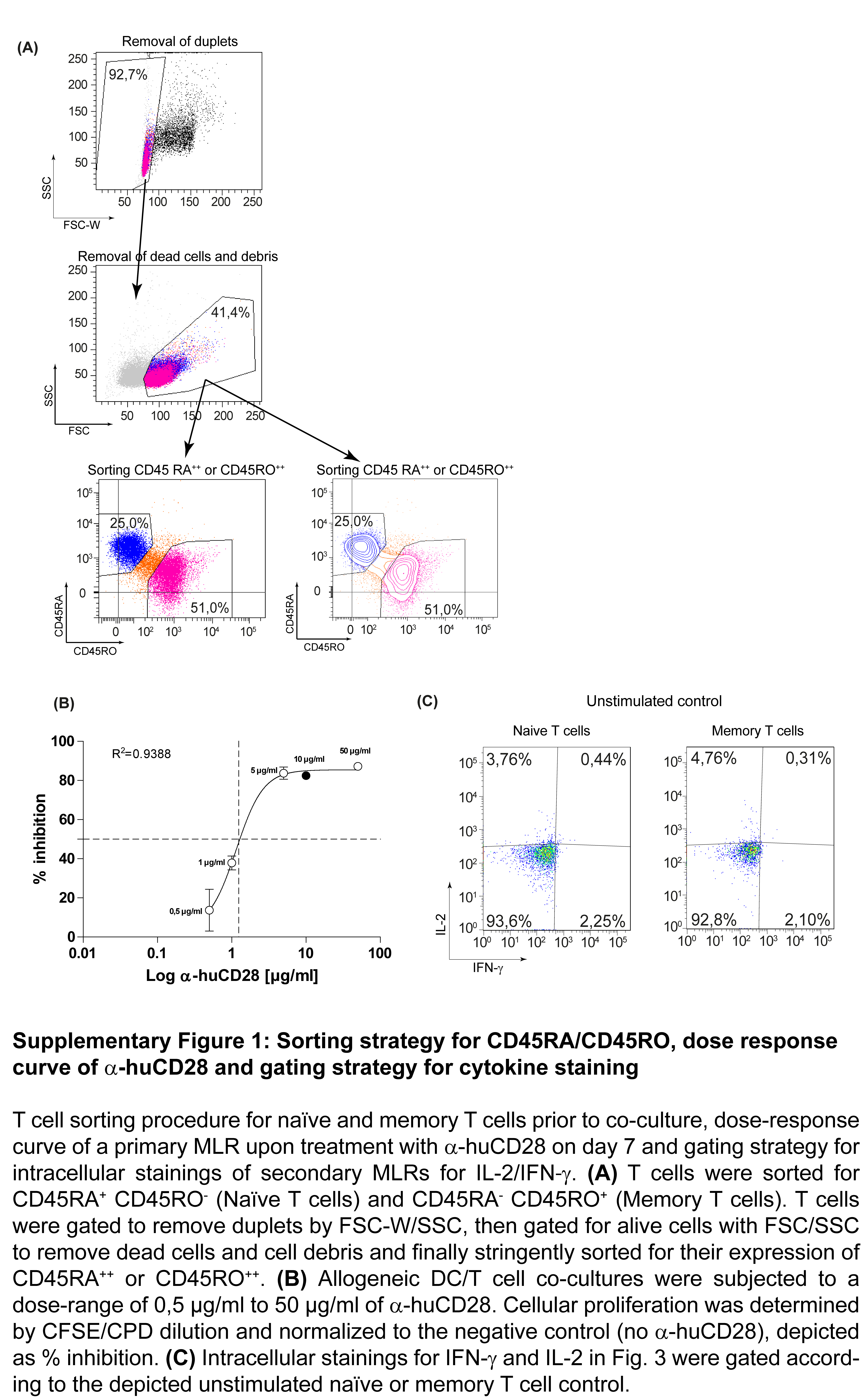

Supplement: Supplementary file 1 [file image_1.tif]

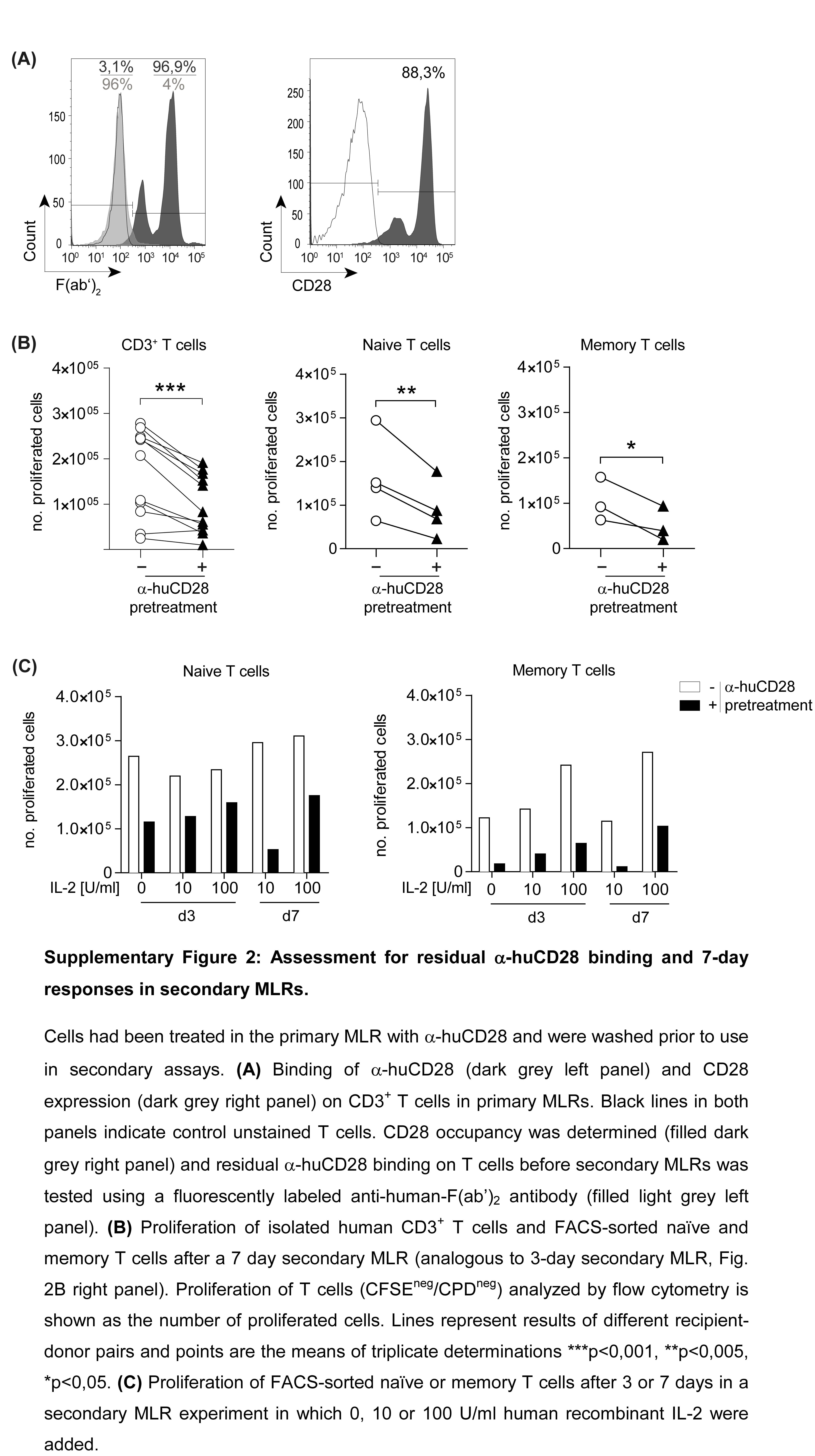

Supplement: Supplementary file 2 [file image_2.tif]

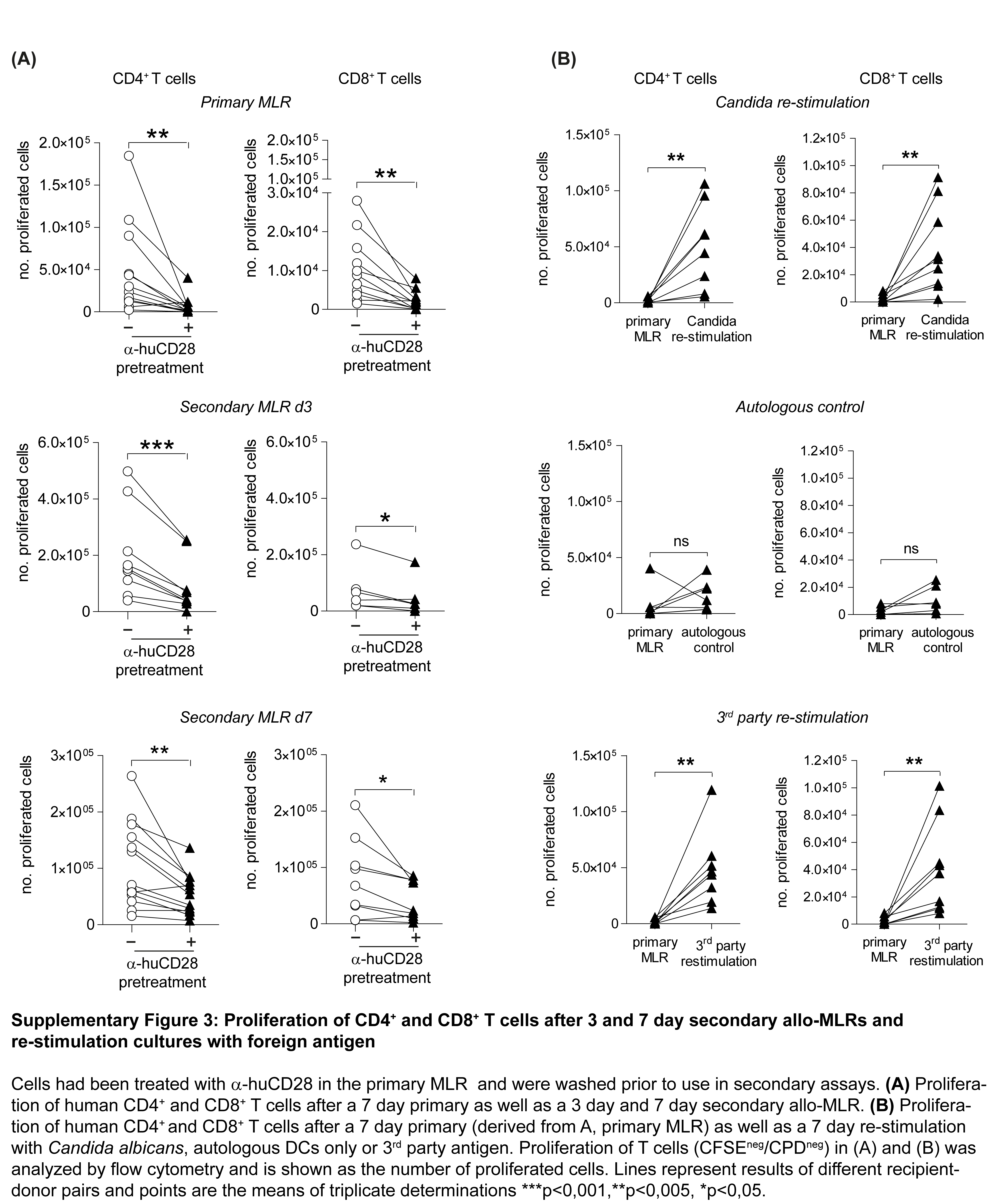

Supplement: Supplementary file 3 [file image_3.tif]

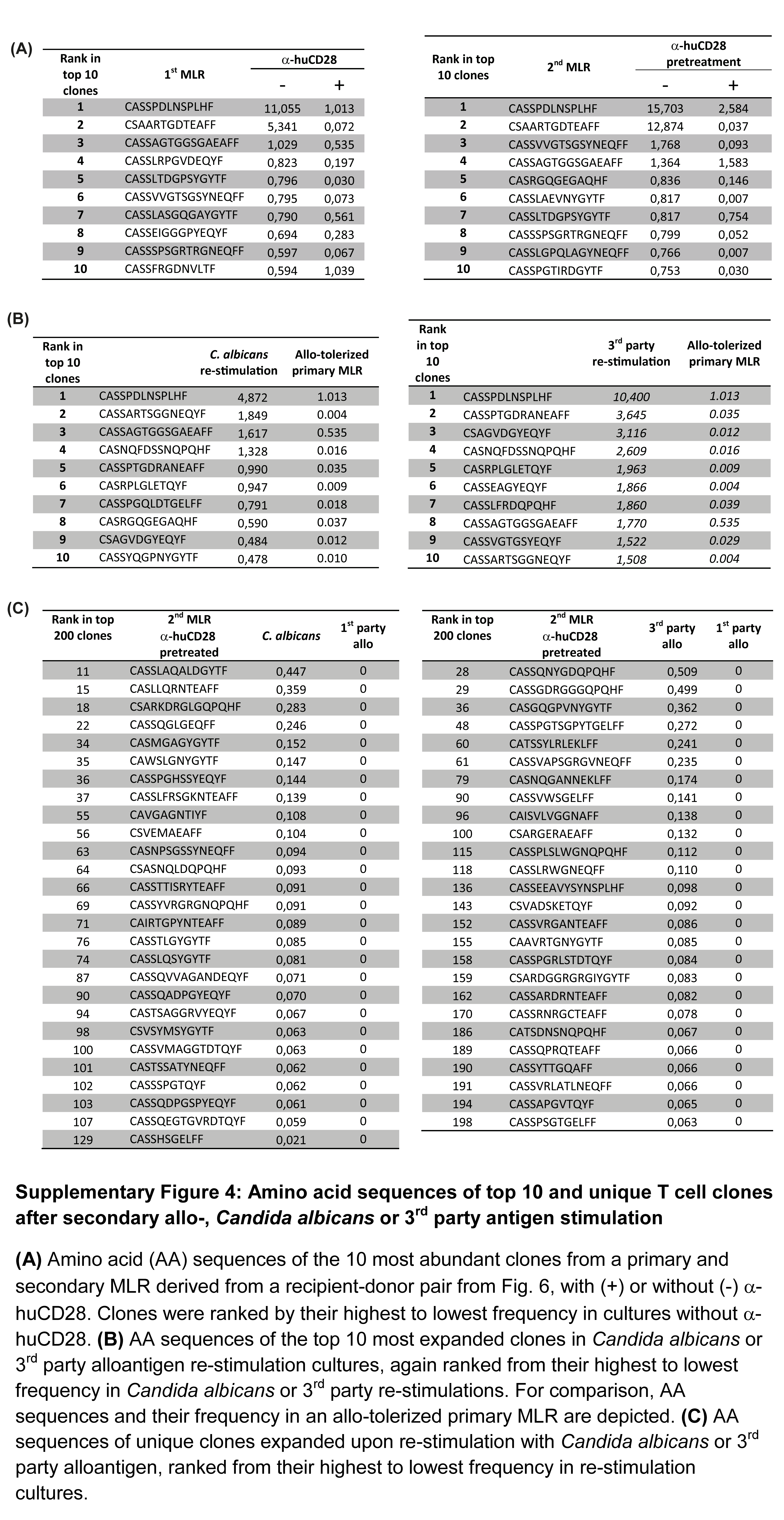

Supplement: Supplementary file 4 [file image_4.tif]

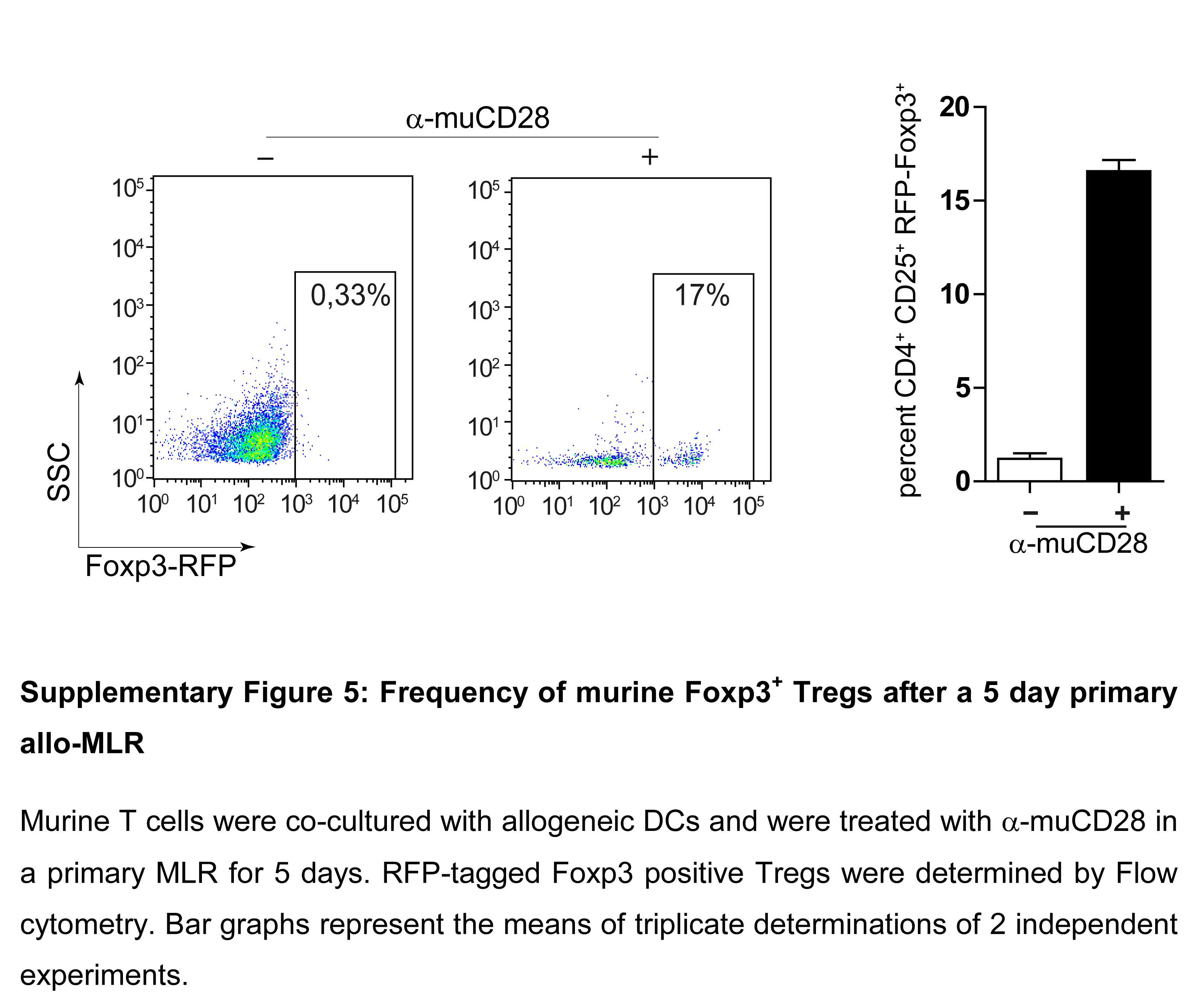

Supplement: Supplementary file 5 [file image_5.tif]
